# Collaborative approaches to health education: perspectives of parents and teachers on self-care and managing common health issues in UK primary schools

**DOI:** 10.1186/s12913-024-11724-3

**Published:** 2024-10-30

**Authors:** Samira Osman, Christine Hirsch, Zahraa Jalal, Vibhu Paudyal

**Affiliations:** 1 School of Pharmacy, Institute of Clinical Sciences, College of Medical and Dental Sciences, University of Birmingham, Birmingham, B15 2TT UK; 2 Florence Nightingale Faculty of Nursing, Midwifery and Palliative Care, King’s College London, London, UK

**Keywords:** Health education, Primary schools, Self-care, Health literacy, Common health issues, Mental well-being, Public health, School-family collaboration.

## Abstract

**Background:**

Health education in primary schools plays a critical role in equipping children with essential self-care skills, fostering health literacy, and addressing social determinants of health. This study explores the perspectives of parents and teachers on teaching self-care and managing common health issues in UK primary schools. Despite the recognized importance of health education, there is limited research on how self-care education is perceived and implemented in the primary school setting.

**Methods:**

A qualitative study was conducted using semi-structured interviews with 18 participants, including 9 generalist primary teachers and 9 parents from diverse educational and socioeconomic contexts in the West Midlands, East Midlands, Northwest, and London of England. Participants were recruited via social media and professional networks, and interviews were conducted via video conferencing platforms. Data were thematically analysed using NVivo 12 to identify recurring themes related to the delivery and impact of self-care education in primary schools.

**Results:**

Teachers and parents highlighted key self-care topics, including hygiene, managing common illnesses like colds and cuts, and mental well-being. There was a consensus on the importance of self-care education, but views diverged on whether the responsibility should lie primarily with schools, parents, or a collaborative approach. Teachers expressed concerns about their preparedness to teach sensitive topics, citing a lack of training and resources. Socioeconomic and religious contexts further influenced perceptions, with participants emphasising further importance of school-led self-care education for children from lower socioeconomic backgrounds. Strengths were identified in current self-care education efforts, particularly in practical lessons that empower children with age-appropriate skills.

**Conclusions:**

This study highlights the critical need for comprehensive and collaborative health education in primary schools to promote health literacy and reduce health disparities. Enhancing teacher training, integrating self-care topics into the primary curriculum, and fostering stronger partnerships between schools and families are essential to improving the quality and consistency of self-care education. Policymakers should consider implementing mandatory health education training in teacher education programs and providing ongoing professional development to support teachers. By addressing these gaps, schools can play a pivotal role in preparing children for lifelong health management, reducing the burden on healthcare services, and supporting public health objectives.

## Background

Health education in primary schools plays a critical role in shaping children’s health behaviours and overall well-being [1]. Unlike early childhood education, which focuses on younger developmental stages, primary education addresses the needs of children typically aged 5–11 years, aligning with key stages 1 and 2 in the UK and Kindergarten- class 6 in the US [2]. This distinction is crucial as it ensures that the educational content is age-appropriate and relevant to the developmental stage of primary school-aged children.

Self-care is defined as “the ability of individuals, families, and communities to promote health, prevent disease, maintain health, and cope with illness and disability with or without the support of a healthcare provider” [3]. In the primary school context, self-care education includes teaching children about hygiene, basic first aid, recognizing symptoms of common health issues, and managing mental health [4]. Empowering children with self-care skills can reduce the burden on healthcare services by enabling them to manage common health issues independently, thereby reducing unnecessary GP consultations [5].

Teaching self-care in primary schools can significantly contribute to developing children’s health literacy, which involves equipping them with the knowledge and skills to make informed health decisions [6]. Health literacy is an essential component of health education that fosters critical thinking and self-regulation, helping children build resilience and confidence in their health management skills [7]. The potential for health education to develop health literacy aligns well with broader public health goals, particularly in addressing social determinants of health that contribute to health disparities [8].

The World Health Organization’s (WHO) Health Promoting Schools Framework emphasizes a whole-school approach to health education, which involves integrating health into all aspects of school life, including the curriculum, school environment, and partnerships with families and the community [9, 10]. This approach is particularly relevant to primary schools, as it supports collaborative efforts to enhance children’s health and well-being, recognizing the influential role of family and community in shaping health behaviours [11].

Schools are uniquely positioned to promote consistent health messages, particularly in communities where family support may be limited due to socioeconomic factors [12, 13]. Incorporating health education in schools can help bridge equity gaps by providing all children, regardless of background, with consistent and reliable health information [14]. This is especially important in low socioeconomic communities where disparities in access to health knowledge and resources can hinder children’s development [15].

Despite the recognized importance of health education, there is limited research in the UK on how self-care and common health issues education are delivered in primary schools and the perspectives of key stakeholders, such as parents and teachers [16–18]. This study aims to address this gap by exploring the views of parents and teachers regarding self-care education for primary school-aged children and its potential impact on public health.

### Interview study aims

The aim of this study is to explore the views of parents and teachers regarding the teaching of self-care and basic health management in UK primary schools. The study seeks to understand how these stakeholders perceive the role of schools in promoting health literacy, managing common health issues, and supporting children’s physical and mental well-being.

#### Research questions

To achieve this aim, the study addressed the following research questions: What are the perceptions of parents and teachers on the role of primary schools in teaching self-care and managing common health issues? And how do parents and teachers perceive the impact of self-care education on children’s physical health (e.g., managing common health issues ) and mental well-being?

3. What are the perceived barriers and strengths in implementing self-care education within primary schools?

## Methods

### Ethics approval

The University of Birmingham Science, Technology, Engineering and Mathematics Ethical Review Committee gave ethical permission for this study, approval number ERN_21-1091 on 18.10.2021. Informed consent was obtained from all participants in the study.

### Study design and participants

This study employed qualitative semi-structured interviews to explore the views of parents and teachers on self-care and common health issues education in primary schools. Interviews were chosen over focus groups because they allow for a more in-depth exploration of individual experiences, particularly on sensitive topics that may be difficult to discuss openly in a group setting [19]. The participants included parents of primary school-aged children and professionals responsible for teaching health, well-being, or physical education subjects in primary schools.

### Demographic context

Participants were recruited from diverse settings, including the West Midlands, East Midlands, Northwest, and London. West Midlands is a region that encompasses both urban and rural areas including some of the most deprived areas in England. In contrast, London, encompassing multiple demographic groups, has a wide range of socioeconomic contexts; however, participants were primarily from inner-city areas known for their socioeconomic diversity.

### Recruitment and data collection

Participants were recruited via personal, professional, and social media networks, including Twitter, LinkedIn, and Facebook, in the UK. This approach facilitated broad outreach and utilized snowball sampling to enhance recruitment. Invitations targeted teaching staff and parents with participants self-selecting by responding voluntarily.

Interviews were conducted via Zoom. Each interview lasted between 30 and 45 min, with a mean duration of approximately 37 min. The interviews were recorded with participant consent, transcribed verbatim, and pseudo- anonymised to protect participant confidentiality. A detailed interview guide (see Table 1) was used to maintain consistency, covering topics such as children’s attitudes towards health, the meaning of self-care, and perceived gaps and barriers in current educational practices.

**Table 1:** Interview schedule

Key Interview Questions
How do you perceive primary school-aged children’s attitudes towards health?
How do you define self-care in the context of the primary school curriculum?
What do you believe should be included in health education within primary schools?
What do you perceive as the gaps and strengths in current self-care education in primary schools?
What barriers do you perceive to implementing effective self-care education in primary schools?
Who do you think is responsible for teaching self-care to primary school children, and why?
What impact do you believe self-care education has or could have on children in primary schools?

### Data analysis

The interview transcripts were coded using NVivo 12, a qualitative data analysis software. Coding was initially performed by the lead researcher (SO) and reviewed by a second researcher (VP) to ensure reliability. Themes were refined through iterative discussions, merging similar codes to capture the essence of participants’ experiences comprehensive which helped to identify recurring themes and sub-themes.

### Demographic results

A total of 18 participants were interviewed, including 9 teaching professionals and 9 parents. The teaching staff consisted of teachers, a dance instructor and an assistant head teacher. All were from primary schools, extracurricular club providers, special educational needs schools and religious school settings specifically it was an Islamic school. Experience ranged from 1 year to more than 20 years.

Four fathers and 5 mothers were interviewed. These were parents of 25 children aged between 2 and 19 years. Table 2 shows the demographic results.

**Table 2:** Demographic results

	Job Role		Sector		Specialism/year group		Gender	Experience
ZT	Teacher		Primary School		Year 3		Female	1–5 years
YT	Dance Instructor		Primary school and private extracurricular club provider		Dance, Year 1–6		Female	15–19 years
ST	Teacher/home educator		Primary school and homeschooling		Year 4		Female	11–14 years
SA	Teacher		Islamic school		Year 3		Male	6–10 years
AH	Teacher		SEN		Year 5/6		Male	20 + years
LB	Teacher		Primary school		Year 4		Male	6–10 years
AG	Assistant Head Teacher		Primary school				Female	6–10 years
SS	Teacher		Primary School		Reception		Female	1–5 years
RF	Teacher		Primary school		Year 2		Male	6–10 years
			Locality		Age of Children			
GM	Father		West midlands		11,7,3			
SE	Mother		West midlands		6,7			
ND	Father		East midlands		7,10			
MK	Mother		London		19,16,15,12,10,4			
EM	Mother		Northwest		9,4			
DP	Mother		West midlands		9,7,5			
BA	Mother		West midlands		6,2			
HA	Father		Northwest		13,8			
HE	Father		London		12,7,5

## Results

The analysis of the interviews identified three main themes: (1) Debates Over Self-Care Teaching Responsibility, (2) Children’s Attitudes Towards Health and Self-Care, and (3) Barriers and Strengths in Self-Care Education. Quotes from participants are used to illustrate key points within each theme, highlighting diverse perspectives based on socioeconomic, cultural, and educational contexts.

### 1. Debates over self-care teaching responsibility

This theme captures the ongoing debate about who holds the primary responsibility for teaching self-care to children—schools, parents, or a shared approach. Most participants acknowledged the importance of self-care education, but opinions varied on who should lead this effort.

Many teachers felt that self-care education should be a shared responsibility between schools and parents. One participant emphasized the need for collaborative approaches: *“It is almost possible as workshops need to be held with both parents and the school. Definitely*,* this is the duty of both the school and parents as well.”* (MK, Father). However, some teachers highlighted practical challenges, such as limited time and resources, which constrain their ability to fully engage in health education: *“We don’t have the time here in the school to give health-related advice; surely parents or health professionals are the right ones for this.”* (LB, Year 4 Teacher, Male).

The role of the school environment was particularly emphasized by participants who felt that children spend a significant amount of time at school, making it an ideal setting for health education: *“Children spend more than half of their lives in schools…everything regarding their health needs to come from school because the main life is about them like the school community.”* (BA, Mother). Conversely, some parents believed that health education should primarily be a parental responsibility, especially when it comes to sensitive topics. One father expressed, *“As a father it’s my duty to look after the children when they are not well; I can’t see how a child would be able to do it themselves.”* (HA, Father).

Cultural and religious contexts also influenced these views. Parents from faith-based schools expressed discomfort with certain health topics being taught in school: *“I think teaching self-care is fine*,* but there are boundaries. I don’t want my child learning about things that conflict with our beliefs.”* (HE, Mother). Another participant added, *“Parents could be a barrier—they might see something on a list and say*,* well*,* I don’t want my child to be taught that; for example*,* a parent may not want their child to be taught sex due to cultural barriers.”* (EM, Mother).

### 2. Children’s attitudes towards health and self-care

Participants described varying levels of engagement and understanding of self-care among children, often shaped by both school and home environments. A teacher noted, *“Self-care starts off with your mindset*,* so it is mentally being in a happy place…physically taking the best care of yourself through nutrition and exercise.”* (YT, Dance Teacher, Female). Practical, hands-on lessons such as hygiene and basic first aid was reported to be particularly engaging for children, with a teacher sharing, *“We looked at how to wrap bandages and different types of injuries… It makes them feel empowered.”* (LB, Year 5 Teacher, Male).

However, participants also highlighted the challenges children face in grasping more abstract health concepts, such as mental well-being. A Year 4 teacher mentioned, *“Self-care for me as well as the children is about taking care of your mental wellbeing…being aware of how to manage stresses and anxieties.”* (ST, Year 4 Teacher and Home Educator, Female). This suggests that while children are eager to engage with practical self-care lessons, there may be gaps in their understanding of mental health, necessitating tailored approaches to teaching these topics.

The maturity and developmental stage of children also influenced their capacity for self-care. One teacher shared, *“It depends on the child…I had one class that was quite mature*,* and the other class was less able to look after themselves and needed more support.”* (RF, Year 2 Teacher, Male). Another teacher expressed concern about children’s awareness of self-care as they grow older: *“In regard to health and self-care…I did not think they are aware of it. As they grow older they are more aware of it.”* (SS, Reception Class Teacher, Female). This variability highlights the importance of age-appropriate and developmentally sensitive approaches to self-care education.

### 3. Barriers and strengths in self-care education

Participants identified several barriers to implementing effective self-care education, including a lack of teacher training, time constraints within the curriculum, and varying levels of parental engagement. One assistant headteacher highlighted the critical need for professional development: *“Most teachers in schools do not have much training and up-to-date knowledge regarding self-care… If training is given to teachers and it is made compulsory then I can see that this can work in schools.”* (AG, Assistant Head Teacher, Female).

Socioeconomic factors also played a significant role, with financial constraints often limiting schools’ capacity to prioritize health education. A mother pointed out, *“Funding is being pulled from schools all the time… the challenges of balancing other subjects with this and the financial costs of bringing in health professionals.”* (SE, Mother). These comments underscore how resource limitations can hinder the integration of self-care education into the school curriculum, particularly in underfunded schools.

Some participants noted that parental attitudes could be a barrier, particularly when parents hold different views on what should be taught: *“Schools may not have the time or resources to teach this*,* and children might have a different strategy at home and a different strategy at school.”* (DP, Mother). However, other participants felt that there were no significant barriers, especially considering recent global health challenges: *“There are no barriers; especially after COVID-19*,* self-care is even more important; we need to teach the children how to look after themselves.”* (AH, SEN Teacher, Female).

Despite these barriers, participants also identified strengths in self-care education. Practical lessons on basic hygiene, managing minor illnesses, and first aid were seen as particularly beneficial. A father emphasized, *“The impact of teaching self-care education is to reduce the burden on teachers and parents after all because when children get this education from school they do not need to ask from parents and teachers as well.”* (EM, Mother). Another participant highlighted the long-term benefits, stating, *“The main impact is that you teach future generations…the right information passes on*,* which is quite important because we only have one body*,* and we need to be taught to look after it.”* (GM, Father).

Some participants recognized the broader implications of self-care education for public health, noting that a well-educated population could reduce the strain on healthcare systems: *“Well*,* you would like to think that you have a healthier society regarding self-care and common health issues …so that people can enjoy as active a life as possible for as long as possible.”* (RF, Reception Class Teacher, Male).

## Discussion

The findings of this study underscore the critical need for comprehensive and collaborative health education in primary schools, linking directly to broader public health objectives. Health literacy, defined as the ability to access, understand, and use information to make health-related decisions, is increasingly recognized as a key factor in promoting long-term health and reducing health disparities [20]. In the UK, where the curriculum includes elements of physical and mental health education, the focus remains on empowering students with knowledge and skills that foster healthy behaviours [21]. However, the delivery of this education is often inconsistent, particularly when taught by generalist primary teachers who may lack the confidence or training required to handle sensitive topics [22].

The importance of health education in schools extends beyond individual knowledge; it plays a significant role in addressing social determinants of health (SDH), including socioeconomic status, access to health resources, and parental engagement [23]. Educators have a responsibility to support students’ health needs, and school-based health education offers an equitable platform to reach all children, including those from less engaged households [24]. Less engaged parents may lack time, knowledge, or resources to reinforce health education at home, often due to socioeconomic pressures or limited health literacy themselves [25]. This highlights the need for schools to take a proactive role in bridging these gaps and providing consistent health messaging that might not be available in every family environment.

The variation in engagement between parents, influenced by factors such as school location and community demographics, aligns with global public health trends [26]. Published research findings show that socioeconomic disparities affect children’s access to health education and resources, reinforcing the necessity for schools to serve as a frontline in public health promotion [27]. For example, children in lower socioeconomic settings may know about healthy eating but lack the means to act on this knowledge due to financial constraints or limited access to healthy food options. School-based health education thus plays a pivotal role in equipping children with the skills and knowledge to navigate health-related decisions, preparing them for future independence [28].

Some participants expressed concerns about whether primary school children are too young to engage with self-care education, particularly around sensitive topics [29]. However, current UK guidelines support age-appropriate health education from the earliest stages of schooling, advocating for a strengths-based approach that empowers students without exposing them to content beyond their developmental capacity [30]. This aligns with the broader salutogenic approach in health education, which emphasizes building resilience and positive health behaviours rather than focusing solely on risk avoidance [31].

The teaching of health education, particularly self-care, is not universally embedded within the initial training of primary teachers in the UK, contributing to a lack of preparedness and confidence among educators [32]. This study’s findings suggest that additional professional development, resources, and policy support are needed to better equip generalist teachers for this role [33]. Internationally, many countries integrate health and physical education into a combined curriculum taught by specialized educators, highlighting a gap in the UK’s approach where primary teachers often manage health education alongside other subject areas [34]. Addressing this through targeted training and clearer curriculum guidance could improve the quality and consistency of health education delivery in schools.

The debate over who should be responsible for teaching self-care—schools, parents, or a shared approach—was a recurring theme in the findings. Children’s capacity for self-care often depends on the level of support they receive at home, suggesting that health education should not only impart knowledge but also address the broader social inequities that influence children’s health outcomes [35]. This requires schools to build stronger collaborative relationships with families, especially those who may be less involved due to socioeconomic barriers [36]. Policies that provide resources and training for schools to engage positively with families could help mitigate these disparities and promote a more synergistic approach to health education.

### Implications for policy and practice

The study highlights the need for integrated, collaborative health education frameworks within the UK primary school curriculum that extend beyond knowledge transmission to address equity gaps in public health. Schools must be equipped with the training, resources, and support necessary to deliver effective health education, especially given the reliance on generalist teachers. Policymakers should consider mandating health education training in teacher education programs and providing ongoing professional development opportunities that align with public health priorities.

Furthermore, schools need support in building relationships with families, particularly those who may be less inclined or able to participate in their child’s health education. Creating stronger school-family partnerships can help reinforce health messages at home, enhancing the overall impact of school-based health education. By addressing both educational gaps and the broader social determinants of health, schools can play a crucial role in fostering health literacy and reducing health inequities among children.

### Limitations

This study was conducted in specific regions of the UK, including the West Midlands, East Midlands, Northwest, and parts of London, which have diverse socioeconomic and demographic characteristics. While this diversity adds depth to the findings, it also limits the generalizability of the results to other contexts.

### Future research directions

Future research could further explore how these contextual factors influence health education practices across different settings. Future studies should explore the impact of specific training interventions on teacher confidence and effectiveness in delivering health education. Comparative research between generalist and specialist educators could provide further insights into optimizing health education delivery in primary schools.

## Conclusion

This study provides valuable insights into the perspectives of parents and teachers on self-care education in UK primary schools, highlighting the critical role that schools play in promoting health literacy and addressing social determinants of health. By examining the teaching of self-care and basic health management, this study fills a significant gap in the literature, where limited research exists on how self-care education is perceived and delivered in the primary school setting. The findings underscore the importance of equipping children with the skills to manage common health issues and support their mental well-being, which is vital for reducing healthcare burdens and promoting long-term health outcomes.

The study also identifies a crucial need for enhanced training and support for primary generalist teachers, who often feel underprepared to teach health topics, especially those that are sensitive or complex. This highlights a gap in current educational practice, where health education is inconsistently delivered due to variations in teacher confidence and training. By bridging this gap through targeted professional development and clearer curriculum guidance, schools can better meet the needs of their students and contribute to closing health equity gaps.

Furthermore, the study emphasizes the value of collaborative approaches that engage both schools and families, advocating for stronger partnerships to ensure consistent health messaging across home and school environments. Addressing these gaps through policy and practice improvements will not only enhance the delivery of self-care education but also support a holistic approach to child health that prepares students for lifelong well-being. This research provides a foundation for future studies to explore more deeply the mechanisms by which school-based health education can impact public health and the broader social determinants of health.

## Data Availability

Data is provided within the manuscript or supplementary information files.
